# Book Review: Basic Law and Ethics for Nursing: Pa-tients, Rights and Decision Making

**DOI:** 10.5195/jmla.2025.2173

**Published:** 2025-07-01

**Authors:** Muhammad Taufan Umasugi

**Affiliations:** 1 umasugi53@gmail.com, STIKes Maluku Husada, Jl. Lintas Seram Kairatu, Seram bagian Barat. Indonesia

*Essential Law and Ethics in Nursing: Patients, Rights, and Decision-Making* by Paul Buka is your key to applying law and ethics in nursing practice. This revised third edition addresses the growing complexities of legal and ethical issues in relation to nursing practice, presenting material primarily targeted toward nursing students and healthcare professionals.

Buka's writing is well organized and clearly explains complex legal and ethical ideas that nurses need to navigate the complicated confines of their work-related roles. Using a plethora of legal cases, ethical theories and practical dilemmas, the book teaches and empowers health care providers to navigate ethical decision-making in their practice.

## SCOPE AND CONTENT

The book offers ten chapters, each addressing a different theme within nursing law and ethics. Chapter 1 presents ethical theories including consequentialism, deontology, and virtue ethics. Readers benefit from an overview of the principles in bioethics and its incorporation in nursing practice.

Later chapters explore more practical aspects of law and ethics in nursing. Chapter 2 examines human rights, including the legal implications for patients of the Human Rights Act 1998 and similar frameworks. Informed consent, the subject of Chapter 5, is further developed through case law including Chester v. Afshar (2004) and Montgomery v. Lanarkshire Health Board (2015). These cases highlight how these legal precedents have come to influence the standards of consent and autonomy as they apply to medical practice in contemporary society, which have meaningful lessons for nurses regarding patient autonomous behaviour.

A chapter addressing professional accountability — Chapter 6 — is, in our opinion, particularly valuable. Buka expertly unpicks ideas such as negligence, duty of care, and the legal underpinnings of professional misconduct, referencing landmark cases such as Bolam v. Friern Hospital Management Committee (1957) and Bolitho v. City and Hackney Health Authority (1997). Dissecting the Bolam test and its development, Buka demonstrates how the law can be demystified within the realm of professional conduct, helping nurses both understand the legalese of the profession and avoid wrongful recourse.

Chapter 7 of the book covers vulnerable health care populations with a focus on elder abuse and mental health care, both areas in which nurses are sometimes faced with balancing ethical principles with legal duties. Another key topic is equality and diversity in healthcare, talked about in Chapter 8. Buka delves into anti-discrimination laws and the importance of cultural competence in delivering equitable care to diverse patient populations.

Chapter 9 broadens the conversation to the ethical dilemmas encountered at the dusk of life, including discussions of euthanasia, palliative care, and ending one's own life. Through cases such as Airedale NHS Trust v. Bland, 1993, Buka observes the ways in which the legal system has shaped decision-making in end-of-life care, highlighting the challenging decisions that must often be made by nurses in these sensitive contexts.

## SIGNIFICANCE AND COMPARISON WITH OTHER WORKS

There are a number of reasons why this book is important. On a more direct note, it fill the gap between learning theory and application. This differs from some other books that might be more theoretical or technical in nature, for example, “Law and Professional Issues in Nursing” by Griffith and Tengnah, but Buka's book shines in its integration of case studies and reflective exercises. This interactive approach, in addition, enhances student and professional engagement and cultivates the critical thinking ability needed for health care ethical decision-making.

Compared with something similar- to Johnstone's Ethics in Nursing Practice which focuses specifically on ethical frameworks that guide nursing practice, Buka's book took a broader approach by intertwining both legal considerations and ethical considerations. The book includes a thorough analysis of particular case law and legal precedent necessary in achieving an understanding of the legal duties owed by nurses and distinguishes this work from other books in this arena.

Although there are many nursing ethics textbooks based on the ethical principles of beneficence, non-maleficence, and autonomy, few come with such a strong legal foundation. The combination of both aspects of law and ethics by Buka ensures that the nurses are able to cater to the legal competencies coming their way along with the ethical competencies to maintain the standards of their professional domain.

## USEFULNESS AND LIMITATIONS

For nursing students, this book is very helpful given that it provides nursing students with an easy-to-understand overview of the complicated world of nursing law and ethics. Instead of being purely theoretical, the content comes to life through case studies and interactive exercises, meaning you will learn how to directly apply the principles in the real world. With its clear organization and easy-to-read prose, students will find complex legal concepts much more digestible.

The book serves as a useful resource for practicing nurses. This keeps them abreast of recent legal developments in their field. The book is UK-focused but that is a limitation. The legal principles explored are applicable to numerous healthcare systems, but the focus on UK law may feel remote to nurses working in jurisdictions outside the United Kingdom. Nurses who are not under these jurisdictions may need to find resources specific to their own laws.

Furthermore, though the text discusses traditional ethical problems in detail, its exploration of new ethical issues (i.e. artificial intelligence in healthcare, telemedicine, pandemic, and the ethical aspects of pandemics in medical choices) could be other opportunities for expansion in subsequent editions. As the healthcare world continues to evolve, it would be prudent to incorporate discussion on these emerging technologies and their ethical implications.

## TARGET AUDIENCE

This book is mainly targeted at nursing students, nursing educators, and practicing nurses. Since the book is structured in a very systematic way, it makes it perfectly suitable for students who want to learn about the multiple legal and ethical issues in healthcare in a very sophisticated yet simple manner. Educators can integrate case studies and interactive exercises into nursing curricula to strengthen the ethical reasoning skills of students. The book serves as a guide to nurses operating in the fine line between law and ethics, particularly within the realms of post-registration education in law and ethics as well as continuing professional development of nurses new to the profession.

While this book will be of most interest to nurses in the UK, the general principles around patient autonomy, professional accountability, and safeguarding are transferable across diverse health and care systems. Nurses in countries with diverse legal systems may find the ethical principles particularly helpful, but should use the book in conjunction with regional legal resources

## CONCLUSION

Essential Law and Ethics in Nursing: Patients, Rights, and Decision-Making by Paul Buka provides us with a comprehensive guide to the essential legal and ethical issues, the types of questions that all nurses should ask themselves on a regular basis. Its unique fusion of legal case studies, ethical theory, and interactive exercises make it an invaluable resource for both students and practicing nurses. Although its legal content is UK-focused, the ethical concepts explored within apply across the globe and is a timely introductory resource for those wanting to understand the challenging and often complex intersection between law, ethics and patient care.

The book's in-depth treatment of landmark legal precedents like those governing informed consent, confidentiality, and negligence ensures that nurses are prepared to make decisions that are both ethically sound and legally compliant. That said, future editions could benefit from a more global approach and a broader treatment of emerging ethical dilemmas posed by new health technologies.

